# The role of a multidisciplinary team in the management of portal hypertension

**DOI:** 10.1186/s12876-020-01203-4

**Published:** 2020-04-03

**Authors:** Yujen Tseng, Lili Ma, Minzhi Lv, Tiancheng Luo, Chengfeng Liu, Yichao Wei, Chu Liu, Ji Zhou, Zhiping Yan, Pengju Xu, Guohua Hu, Hong Ding, Yuan Ji, Shiyao Chen, Jian Wang

**Affiliations:** 1 Department of Gastroenterology, Zhongshan Hospital, Fudan University, 180 Fenglin Road, Shanghai, 0086200032 China; 2 Department of Digestive Diseases, Huashan Hospital, Fudan University, Shanghai, China; 3 Department of Endoscopy Center, Zhongshan Hospital, Fudan University, Shanghai, China; 4 Department of Biostatistics, Zhongshan Hospital, Fudan University, Shanghai, China; 5 Evidence-based Medical Center, Fudan University, Shanghai, China; 6 Department of Geriatrics, Zhongshan Hospital, Fudan University, Shanghai, China; 7 Department of Interventional Radiology, Zhongshan Hospital, Fudan University, Shanghai, China; 8 Department of Radiology, Zhongshan Hospital, Fudan University, Shanghai, China; 9 Department of General Surgery, Zhongshan Hospital, Fudan University, Shanghai, China; 10 Department of Diagnostic Ultrasound, Zhongshan Hospital, Fudan University, Shanghai, China; 11 Department of Pathology, Zhongshan Hospital, Fudan University, Shanghai, China

**Keywords:** Portal hypertension, Multidisciplinary team, Individualized treatment

## Abstract

**Background:**

Gastroesophageal variceal hemorrhage is the most severe complication of portal hypertension, with a high mortality rate. The current recommendations for gastroesophageal varices include pharmacological treatment, endoscopic treatment, transjugular intrahepatic portosystemic shunt (TIPS) placement, and splenectomy with devascularization surgery. Multidisciplinary team (MDT) comprises of a group of medical experts and specialists across a range of disciplines, providing personalized and targeted patient care tailored to each individual’s condition, circumstances, and expectations.

**Methods:**

Patients referred to the MDT clinic since its establishment in September 2014 were prospectively enrolled and followed-up for at least 12 months. Patient baseline characteristics, treatment methods, outcome and survival were compared to non-MDT patients retrieved from a prospectively maintained database with propensity score matching.

**Results:**

Propensity-score matching (PSM) was carried out to balance available covariates, resulting in 58 MDT patients vs. 111 non-MDT patients. Overall survival and variceal rebleed was compared between the two groups. The rate of variceal rebleed was significantly higher in the non-MDT group, while no difference in overall survival was observed.

**Conclusions:**

This study is the first to investigate the role of a multidisciplinary team in the management of gastroesophageal varices secondary to portal hypertension. Patients treated based on MDT clinic recommendations had a significantly lower risk for variceal rebleed.

## Background

Portal hypertension is associated with a series of clinical presentation such as hepatic encephalopathy, ascites, spontaneous bacterial peritonitis, gastroesophageal varices, and hepatorenal syndrome. Gastroesophageal variceal hemorrhage is the hallmark and arguably the most severe complication of portal hypertension, with a mortality rate of 10–20% within 6 weeks [1]. Despite adequate treatment and resuscitation, rate of variceal rebleed within the first year remains concerning [2, 3]. The current recommendations for gastroesophageal varices include pharmacological treatment as primary prophylaxis, as well as endoscopic treatment with band ligation or tissue adhesive injection. While, the utility of transjugular intrahepatic portosystemic shunt (TIPS) placement may be suitable in high-risk patients [4]. Splenectomy with devascularization surgery is also an option for gastroesophageal varices, especially in patients with concurrent hypersplenism [5].

Multidisciplinary team (MDT) comprises of a group of medical experts and specialists across a range of disciplines, working together to deliver comprehensive patient care [6]. Regardless of guideline recommendations, MDT clinic provides personalized and targeted patient care tailored to each individual’s condition, circumstances, and expectations [7]. Relevant experts with different expertise are brought together to discuss and formulate a treatment plan. The present study is aimed at evaluating the value of MDT clinic in the management of gastroesophageal varices secondary to portal hypertension.

## Methods

### Multidisciplinary team for portal hypertension

The multidisciplinary team (MDT) for portal hypertension at our institution was first established in 2014. The team is comprised of 5 different clinical specialties, including gastroenterology, interventional radiology, general surgery, diagnostic radiology, diagnostic ultrasound, and consulting pathology. Through a multidisciplinary approach, all patients referred to the MDT clinic will receive a recommendation for individualized treatment based on the patient’s disease status, laboratory and imaging studies, as well as personal preference. The MDT clinic is routinely scheduled every 2 weeks at a designated conference room. An example of case management by the MDT clinic is presented in Supplementary 1.

### MDT patients

Patients referred to the MDT clinic since its establishment in September 2014 were eligible for study inclusion, in accordance with the following criteria: 1) patient with a confirmed diagnosis of gastroesophageal varices secondary to portal hypertension, evident by variceal bleeding or endoscopic examination, 2) diagnosis of portal hypertension was confirmed by imaging or pathological studies, 3) patients with complete documentation of the MDT clinic recommendations, including relevant biochemical, radiological, and procedural data. Exclusion criteria were as follows: 1) patients with acute active variceal hemorrhage, or clinically and hemodynamic unstable, 2) patients who failed to comply with recommended therapy, 3) patient with severe, life-threatening disease of another system, irrelevant to portal hypertension and its complications.

### Non-MDT patients

Patient data were retrieved from a prospectively maintained database, with detailed documentation of general characteristics, treatments received, and follow-up for rebleed and mortality. Usage of the database was approved by the Ethics Committee at our institution (B2015-135R).

### Treatment

Different treatment strategies include currently available options for the management of gastroesophageal varices secondary to portal hypertension, including endoscopic therapy, interventional radiology, and surgery. An informed consent was signed by all patients prior to any treatment procedures, acknowledging the purpose and associate risk.

### Endoscopy

Endoscopy treatment for gastroesophageal varices was commenced after an overnight fast. First, the extent and characteristics of the varices were evaluated and classified according to Sarin’s classification with a standard esophagogastroduodenoscopy (Olympus GIF-XG240/260). After a thorough examination, each patient received appropriate personalized therapy as deemed fit by the operator. Esophageal varices were treated with endoscopic band ligation (EBL) (6 Shooter, Universal Saeed, Multi-Band Ligator, Cook Medical or Speedband Superview Super 7, Multiple Band Ligators, Boston Scientific) or endoscopic injection sclerotherapy (EIS). Concurrent or isolated gastric varices were uniformly treated with cyanoacrylate injection, which begins with an injection of lauromacrogol (Tianyu Pharmaceutical, Zhejiang, China), followed by N-butyl-cyanoacrylate (Beijing Suncon Medical Adhesive Co. Ltd., Beijing, China), then again with a flush of lauromacrogol. The amount of lauromacrogol and cyanoacrylate injected directly correlated with the size of the varix. The operator aimed to eradicate the varices in one session, prompting multiple injection sites, when necessary. The needle sheath (Olympus NM-200 L-0423 injection needle) was held at the puncture site until the varix solidified, turned pale, and became less mobile. Patients were hospitalized for 1–2 days after the procedure barring any complications and were followed-up closely at a designated out-patient service clinic.

### Interventional radiology

Interventional radiological treatment modalities for portal hypertension include transjugular intrahepatic portosystemic shunt (TIPS), percutaneous transhepatic variceal embolization (PTVE), total or partial splenic embolization (TSE or PSE), transarterial embolization (TAE) or a combination of the above-mentioned procedures.

The most frequently performed and well-accredited procedure for the treatment of portal hypertension is the TIPS procedure. In brief, the patient was placed in a supine position after an overnight fast. Access to the left branch of the intrahepatic portal vein was achieved with a 21G Chiba needle (Cook, USA) under ultrasound guidance or direct puncture. The right hepatic vein or hepatic segment of the inferior vena cava was chosen for TIPS needle insertion to the branch of the portal vein (typically left branch). An 8 mm × 40 mm balloon is used to dilate the shunt tract and an 8-10 mm × 60-100 mm Smart self-expanding stent (Cordis, USA) or a nitinol self-expanding stent (Bard, USA) was placed. PTVE can also be performed alongside the TIPS procedure in patients with large gastric varices. The catheter is extended to the gastric coronary vein for visualization of the gastric varices. N-butyl-cyanoacrylate (Beijing Suncon Medical Adhesive Co. Ltd., Beijing, China) is injected for embolization. Amount of tissue adhesive used ranged from 0.5-2 ml, depending on the size of the varix. Another procedure commonly performed in patients with hypersplenism is TSE or PSE, which entails the complete or partial embolization of the splenic artery with 300–500 Embosphere (Merit Medical, UT, USA). In patients with hepatic artery-portal vein arteriovenous fistula, TAE is achieved with injection of N-butyl-cyanoacrylate to embolize the fistula tract. All interventional radiological procedures are performed alone or in conjunction with other procedures, depending on the patient’s condition.

### Surgery

The Hassab’s procedure begins with a 15 cm incision along the left rectus abdominus, followed by ligation of the splenogastric ligament, then the pancreatic tail is dissected for exposure of the splenic artery. A double layer surgical stich is used for splenic artery ligation, before removal of the spleen. The procedure is concluded with suture ligation of the left gastroepiploic artery, vascular branch along the gastric corpus and cardia, and a perhiatal devascularization of the esophagus. Hemostasis was confirmed before abdominal closure.

### Other clinical assessment

All patient from the MDT group received a computed tomography angiography (CTA) study of the portal system prior to treatment intervention. This allows for a thorough evaluation for any concurrent conditions such has hepatocellular carcinoma (HCC), portovenous thrombosis (PVT), or spontaneous portosystemic shunt (SPSS), which are common complications associated with portal hypertension. Some of the non-MDT patients also had available portosystemic CTA that were retrospectively reviewed. During the MDT clinic, radiology studies were carefully reviewed by the experts on Centricity Enterprise Web V3.0 (GE Healthcare, Illinois, USA). Some patients from both MDT and non-MDT groups underwent HVPG measurements to better assess disease severity. HVPG measurement was carried out in all MDT patients barring any contraindications. However, it is not a pre-requisite procedure before treatment.

### Patient follow-up

Patients were prospectively followed for at least 12 months in order to document any incidence of variceal rebleed, evident by melena or hematemesis, confirmed by a subsequent endoscopic examination. Primary endpoints were defined as variceal rebleed or death. A thorough review of each subject’s patient history was conducted. Any missing information was obtained through a telephone interview or at the designated outpatient service clinic.

### Propensity score matching

In order to better assess the role of MDT clinic is the management of patients with portal hypertension, a propensity score match (PSM) was carried out to compare MDT patients with non-MDT patients. The propensity score model of MDT was constructed by using the multivariable logistic regression model, which included age, gender, Child-Pugh classification, etiology of portal hypertension, previous treatment, treatment, portal venous thrombosis, hepatic cellular carcinoma and Sarin’s classification. Propensity scores were then matched with a maximal range of ±0.2 to obtain matched pairs of patients. Patients of MDT group were matched at 1:2 to non-MDT patients.

### Statistical analysis

Continuous normally distributed variables are presented as mean ± standard deviation and group comparisons are tested using the Student t test. Ordinal and non-normally distributed variables are presented by median (Q1~Q3) and group comparisons are tested by using nonparametric Wilcoxon rank-sum test. Categorical variables are presented as absolute values (percentage) and group comparisons are tested by using Pearson *X*^*2*^ tests or Fisher exact test.

For the primary endpoint (rebleeding), Kaplan-Meier curves show the cumulative event-free survival rates for each group and were compared by using the log-rank test. To assess the association between rebleeding and the different groups (MDT group vs. non-MDT group), a propensity score matched and an unmatched Cox proportional hazards models were constructed to calculate the hazard ratios (HR). The inverse probability of treatment weight (IPTW) was calculated on the basis of the propensity score. All hypothesis tests were two-sided, and values of < 0.05 were considered statistically significant. All data were analyzed with the R software, version 3.5.1.

## Results

### MDT patients

A total of 120 patients were referred to the MDT clinic from September 2014 through September 2017. Among which, 29 patients failed to comply with recommended treatment strategy due to personal or financial reasons, 2 patients had comorbid myelodysplastic syndrome (MDS), which preceded the treatment of portal hypertension. A total of 89 patients received treatment according to MDT recommendation, however, 3 were lost to follow-up. A final of 86 patients were included in the present study analysis. Of the 86 subjects, 59 (68.6%) were male, 27 (31.4%) were female, with an average ae of 52.91 ± 13.08 years old. 49 (57%) patients were admitted for initial treatment of gastroesophageal varices, while 37 (43%) patients had received previous treatment, including 25 cases of previous endoscopic therapy, 8 cases of previous splenectomy, and 3 cases of PSE, and 1 case of PTVE. The cause of portal hypertension for most patients were mainly due cirrhosis with varying etiologies. The most common cause was viral hepatitis (53.5%), including both hepatitis B virus and hepatitis C virus. Other etiologies included drug or alcohol-induced (4.7%), autoimmune hepatitis or primary biliary cirrhosis (5.8%), schistosomiasis (10.5%), others (22.1%) or mixed etiology (3.5%). The average Child-Pugh score in the MDT group was 6.59 ± 1.32. With most patients classified as Child-Pugh Class A (51.2%), followed by Child-Pugh Class B (47.7%) and Child-Pugh Class C (1.2%). Endoscopic examination revealed esophageal varices (EV) in 16 patients (18.6%), gastroesophageal varices (GOV) type 1 in 23 patients (26.7%), GOV type 2 in 30 patients (34.9%), and isolated gastric varices (IGV) type 1 in 17 patients (19.8%). There are no IGV type 2 in this cohort. Concurrent conditions such as HCC was observed in 9 (10.5%) patients, while PVT was observed in 33 (38.4%) patients. After MDT discussion, 12 (14%) patients received drug therapy, 28 (32.6%) patients received endoscopic treatment, 7 (8.1%) patients received surgery, 37 (43.0%) patients received interventional radiology, and 2 (2.3%) patients received combined treatment. Detailed patient characteristics are shown in Table 1.

**Table 1:** Baseline characteristics of the general population, MDT group and non-MDT group

	Global Population (*n* = 528)	MDT (*n* = 86)	Non-MDT (*n* = 442)
General Characteristics			
Gender			
Male	351 (66.5%)	59 (68.6%)	292 (66.1%)
Female	177 (33.5%)	27 (31.4%)	150 (33.9%)
Age (years)	53.51 ± 10.99	52.91 ± 13.08	53.63 ± 10.55
Child Pugh Score	6.70 ± 1.45	6.59 ± 1.32	6.72 ± 1.47
Child Pugh Classification			
Class A	268 (50.8%)	44 (51.2%)	224 (50.7%)
Class B	240 (45.5%)	41 (47.7%)	199 (45%)
Class C	20 (3.8%)	1 (1.2%)	19 (4.3%)
MELD Score	6.65 ± 4.29	7.04 ± 4.59	6.57 ± 4.23
HVPG (*n* = 135)	13.78 ± 7.33	12.96 ± 7.10	15.04 ± 7.56
Etiology of Portal Hypertension			
HBV	290 (54.9%)	44 (51.2%)	246 (55.7%)
HCV	10 (1.9%)	2 (2.3%)	8 (1.8%)
Alcohol	44 (8.4%)	4 (4.7%)	40 (9%)
PBC	4 (0.8%)	2 (2.3%)	2 (0.5%)
AIH	55 (10.4%)	3 (3.5%)	52 (11.8%)
Schistosomiasis	28 (5.3%)	9 (10.5%)	19 (4.3%)
Cryptogenic	68 (12.9)	6 (7.0%)	62 (14%)
NAFLD	1 (0.2%)	0 (0%)	1 (0.2%)
Others	16 (3.0%)	13 (15.1%)	3 (0.7%)
Mixed	12 (2.3%)	3 (3.5%)	9 (2%)
Laboratory Parameters			
Hemoglobin (g/L)	86.50 (72.00–104.00)	93.50 (76.25–117.75)	88.31 85 (71.00–102.00)
Platelet (× 10^9^/L)	61.00 (43.00–83.25)	65 (39.25–114.75)	61.00 (44.00–80.00)
White blood cell (× 10^9^/L)	2.74 (2.03–3.80)	2.83 (2.02–3.89)	2.73 (2.03–3.78)
Total Bilirubin (μmol/L)	14.55 (10.58–21.50)	14.25 (10.43–21.7)	14.65 (10.63–21.48)
ALT (U/L)	24.00 (16.85–36.00)	19.50 (15.00–27.00)	25.00 (17.00–40.00)
AST (U/L)	32.00 (24.00–44.00)	29.00 (21.25–38.75)	33.00 (24.55–44.00)
Albumin (g/L)	34.00 (30.00–38.00)	36.00 (32.00–40.00)	34.00 (30.00–38.00)
Serum Creatinine (μmol/L)	67.00 (57.00–79.00)	67.00 (58.00–77.75)	67.00 (57.00–79.53)
Prothrombin Time (s)	14.10 (13.10–15.40)	13.80 (12.93–15.25)	14.10 (13.20–15.40)
International Normalized Ratio (INR)	1.22 (1.14–1.33)	1.23 (1.14–1.35)	1.22 (1.13–1.33)
Received Previous Treatment	132 (64.5%)	37 (43.0%)	95 (21.5%)
Concurrent Conditions			
Portal Venous Thrombosis			
Absent	431 (81.6%)	53 (61.6%)	351 (79.4%)
Present	97 (18.4%)	33 (38.4%)	91 (20.6%)
Hepatic Cellular Carcinoma			
Absent	490 (92.8%)	77 (89.5%)	413 (93.4%)
Present	38 (7.2%)	9 (10.5%)	29 (6.6%)
Ascites			
Absent	253 (47.9%)	45 (52.3%)	208 (47.1%)
Mild	232 (43.9%)	40 (46.5%)	192 (43.5%)
Severe	43 (8.1%)	1 (1.2%)	42 (9.5%)
Endoscopic Examination			
Gastroesophageal Classification			
EV	149 (28.2%)	16 (18.6%)	133 (30.1%)
GOV Type 1	298 (56.4%)	23 (26.7%)	275 (62.2%)
GOV Type 2	48 (9.1%)	30 (34.9%)	18 (4.1%)
IGV Type 1	33 (6.3%)	17 (19.8%)	16 (3.6%)
Treatment Received			
Pharmacological Treatment	20 (3.8%)	12 (14%)	8 (1.8%)
Endoscopy	403 (76.3%)	28 (32.6%)	375 (84.8%)
Surgery	55 (10.4%)	7 (8.1%)	48 (10.9%)
Interventional Radiology	45 (8.5%)	37 (43%)	8 (1.8%)
Combined Therapy	5 (9.0%)	2 (2.3%)	3 (0.7%)
Follow-up			
Variceal Rebleed	156 (29.5%)	7 (8.1%)	149 (33.7%)
Time to Rebleed	521.40 (299.75–989.70)	382.50 (289.50–508.25)	579.30 (301.35–1062.60)
Mortality	86 (16.3%)	9 (10.5%)	77 (17.4%)
Time to Death	728.25 (420.68–1198.58)	382.50 (289.50–508.25)	843.15 (467.40–1368.00)

### Non-MDT patients

Patient data were retrieved from a prospectively maintained database. Prior to the establishment of MDT clinic, patients received at different outpatient clinic underwent standardized care. A total of 462 patients with gastroesophageal varices secondary to portal hypertension treated from August 2008 through August 2014 were retrospectively reviewed. Twenty patients were lost to follow-up within 12 months. A total of 442 patients were ultimately included for statistical analysis. Among which, 292 (66.1%) were male and 150 (33.9%) were female, with an average age of 53.63 ± 10.55. Three hundred forty-seven (78.5%) patients were admitted for initial treatment of gastroesophageal varices, while 95 (21.5%) patients had received previous treatment. The most common etiology for portal hypertension was cirrhosis secondary to viral hepatitis (57.5%). While drug or alcohol-induced cirrhosis accounted for 9.0%, autoimmune hepatitis or PBC for 12.2%, schistosomiasis for 4.3%, others for 14.9%, and mixed etiology for 2.0%. The average Child-Pugh score was 6.72 ± 1.47, with 224 (50.7%) patients classified as Child-Pugh Class A, 199 (45%) classified as Child-Pugh Class B, and 19 (4.3%) classified as Child-Pugh Class C. Endoscopic examination showed 133 (30.1%) cases of EV, 275 (62.2%) cases of GOV type 1, and 18 (4.1%) cases of GOV type 2, 16 (3.6%) cases of IGV type 1. No IGV type 2 was noted. Concurrent HCC was observed in 29 (6.6%) patients, while PVT was observed in 91 (20.6%) patients. Standardized care dictated 8 (1.8%) cases of pharmacological treatment, 375 (84.8%) cases of endoscopic therapy, 48 (10.9%) cases of surgery, 8 causes (1.8%) cases of interventional radiology, and 3 (0.7%) cases of combined treatment. Detailed patient characteristics are shown in Table 1.

### Propensity score matching

**Fig. 1:**
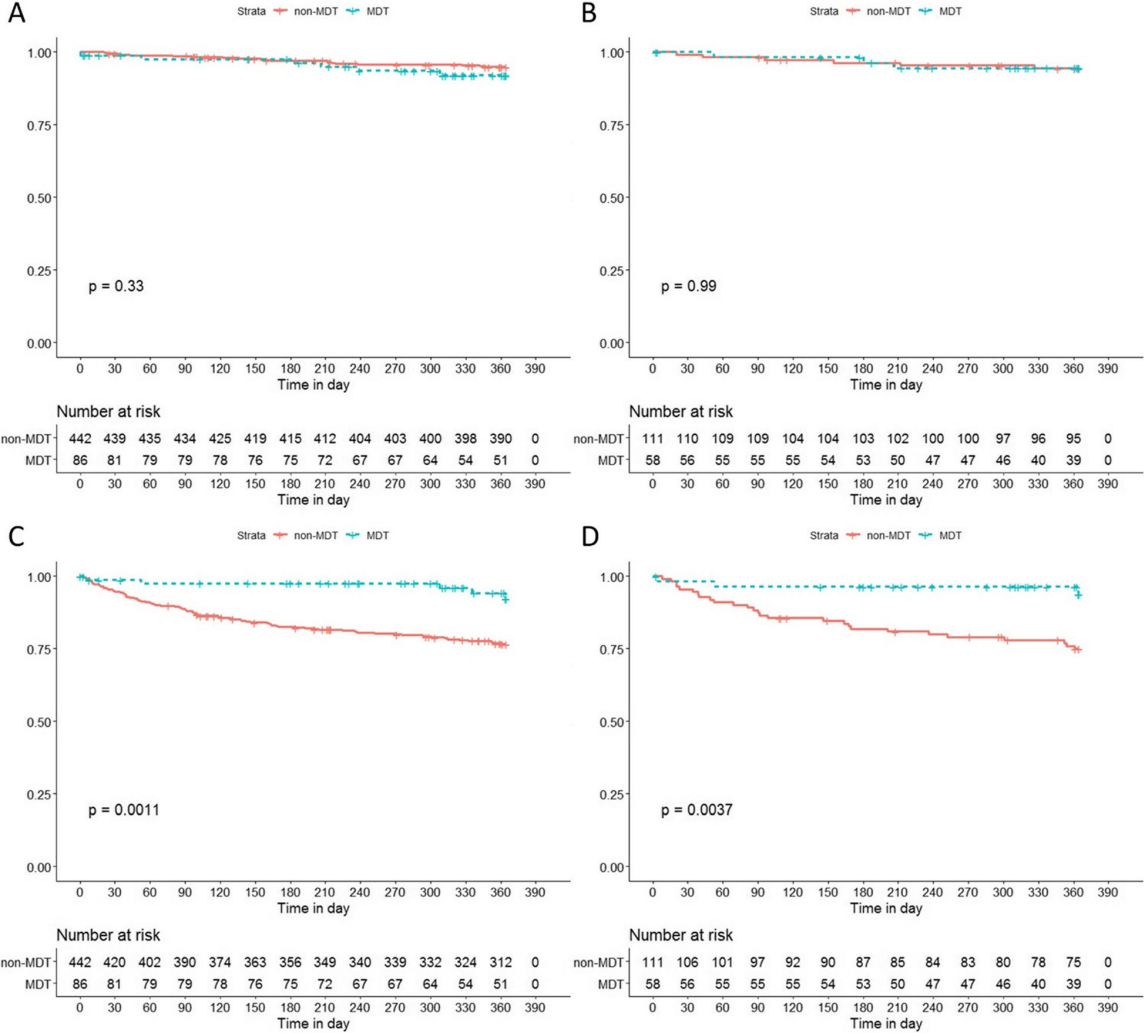
Kaplan-Meir analysis of survival (**a**) (**b**) and variceal rebleeding (**c**) (**d**), before and after PSM

The following variables were selected for propensity score matching (PSM): age, gender, previous treatment, treatment type, etiology of portal hypertension, concurrent hepatocellular carcinoma (HCC), concurrent portal venous thrombosis (PVT), Child-Pugh score, and Sarin’s classification. Before PSM, no significant statistical difference between age, gender, Child-Pugh score, and concurrent HCC was observed between MDT and non-MDT patients. However, statistical difference was noted in previous treatment, treatment method, etiology of portal hypertension, presence of PVT, and type of gastroesophageal varices (Table 2). PSM was carried out to balance almost all available covariates, resulting in 58 MDT patients vs. 111 non-MDT patients. Based on the priori established primary endpoints, overall survival and variceal rebleed was compared between the two groups. The rate of variceal rebleed was significantly higher in the non-MDT group, while no difference in overall survival was observed (Fig. 1). The actuarial rate of no variceal rebleed at 1-month, 2-months, 3-months, 6-months, and 1-year for the non-MDT group, was 95.5, 91.0, 87.4, 81.8, and 75.0%, while that of the MDT group was 98.2, 96.5, 96.5, 96.5, and 93.9%, respectively (Table 3). The Kaplan-Meier analysis indicated that MDT group patients have a lower rate of variceal rebleed within 1 year. Cox proportional hazard regression analysis revealed that patients treated based on MDT clinic recommendation had a lower risk of variceal rebleed (hazard ratio 0.203, 95% CI 0.062–0.670, *p* < 0.01), while IPTW coincided similar results (hazard ratio 0.106, 95% CI 0.026–0.436, p < 0.01).

**Table 2:** Baseline characteristics of the MDT and non-MDT groups, before and after PSM

	Global Population (*n* = 528)			Propensity Score Matching (*n* = 169)		
	MDT (*n* = 86)	Non-MDT (*n* = 442)	*P*-value	MDT (*n* = 58)	Non-MDT (*n* = 111)	*P*-value
General Characteristics						
Gender						
Male	59 (68.6%)	292 (66.1%)	0.74	39 (67.2%)	88 (79.3%)	0.275
Female	27 (31.4%)	150 (33.9%)		19 (32.8%)	23 (20.7%)	
Age (years)	52.91 ± 13.08	53.63 ± 10.55	0.579	51.76 ± 12.39	50.68 ± 11.73	0.089
Child Pugh Classification						
Class A	44 (51.2%)	224 (50.7%)	0.371	28 (48.3%)	55 (49.5%)	0.761
Class B	41 (47.7%)	199 (45%)		29 (50.0%)	52 (46.8%)	
Class C	1 (1.2%)	19 (4.3%)		1 (1.7%)	4 (3.6%)	
MELD Score	7.04 ± 4.59	6.57 ± 4.23	0.359	7.51 ± 4.09	7.22 ± 3.78	0.64
Etiology of Portal Hypertension						
Viral Hepatitis	46 (53.5%)	254 (57.5%)	0.027	35 (60.3%)	60 (54.1%)	0.894
Alcohol	4 (4.7%)	40 (9%)		2 (3.4%)	8 (7.2%)	
AIH/PBC	5 (5.8%)	54 (12.2%)		4 (6.9%)	7 (6.3%)	
Schistosomiasis	9 (10.5%)	19 (4.3%)		6 (10.3%)	10 (9.0%)	
Others	19 (22.1%)	66 (14.9)		10 (17.2%)	23 (20.7%)	
Mixed	3 (3.5%)	9 (2%)		1 (1.7%)	3 (2.7%)	
Received Previous Treatment	37 (43.0%)	95 (21.5%)	< 0.001	30 (51.7%)	53 (47.7%)	0.742
Concurrent Conditions						
Portal Venous Thrombosis						0.841
Absent	53 (61.6%)	351 (79.4%)	0.005	52 (89.7%)	102 (91.9%)	
Present	33 (38.4%)	91 (20.6%)		6 (10.3%)	9 (8.1%)	
Hepatic Cellular Carcinoma						0.886
Absent	77 (89.5%)	413 (93.4%)	0.292	50 (86.2%)	98 (88.3%)	
Present	9 (10.5%)	29 (6.6%)		8 (13.8%)	13 (11.7%)	
Endoscopic Examination						
Gastroesophageal Classification						
EV	16 (18.6%)	133 (30.1%)	< 0.001	14 (24.1%)	35 (31.5%)	0.158
GOV Type 1	23 (26.7%)	275 (62.2%)		19 (32.8%)	46 (41.4%)	
GOV Type 2	30 (34.9%)	18 (4.1%)		16 (27.6%)	16 (14.4%)	
IGV Type 1	17 (19.8%)	16 (3.6%)		9 (15.5%)	14 (12.6%)	
Treatment Received						
Pharmacological Treatment	12 (14%)	8 (1.8%)	< 0.001	8 (13.8%)	8 (7.2%)	0.004
Endoscopy	28 (32.6%)	375 (84.8%)		26 (44.8%)	68 (61.3%)	
Surgery	7 (8.1%)	48 (10.9%)		7 (12.1%)	24 (21.6%)	
Interventional Radiology	37 (43%)	8 (1.8%)		15 (25.9%)	8 (7.2%)	
Combined Therapy	2 (2.3%)	3 (0.7%)		2 (3.4%)	3 (2.7%)

**Table 3:** Difference in rate of variceal rebleed between MDT and non-MDT groups

	Before PSM		After PSM	
	MDT	non-MDT	MDT	non-MDT
1-month actuarial survival	0.976 (0.943–1)	0.907 (0.881–0.935)	0.982 (0.949–1)	0.955 (0.917–0.994)
2-month actuarial survival	0.976 (0.943–1)	0.885 (0.855–0.915)	0.965 (0.918–1)	0.91 (0.858–0.965)
3-month actuarial survival	0.976 (0.943–1)	0.825 (0.79–0.861)	0.965 (0.918–1)	0.874 (0.814–0.938)
6-month actuarial survival	0.923 (0.858–0.992)	0.765 (0.726–0.806)	0.965 (0.918–1)	0.818 (0.749–0.894)
1-year actuarial survival	0.976 (0.943–1)	0.907 (0.881–0.935)	0.939 (0.873–1)	0.75 (0.672–0.836)

**Fig. 2:**
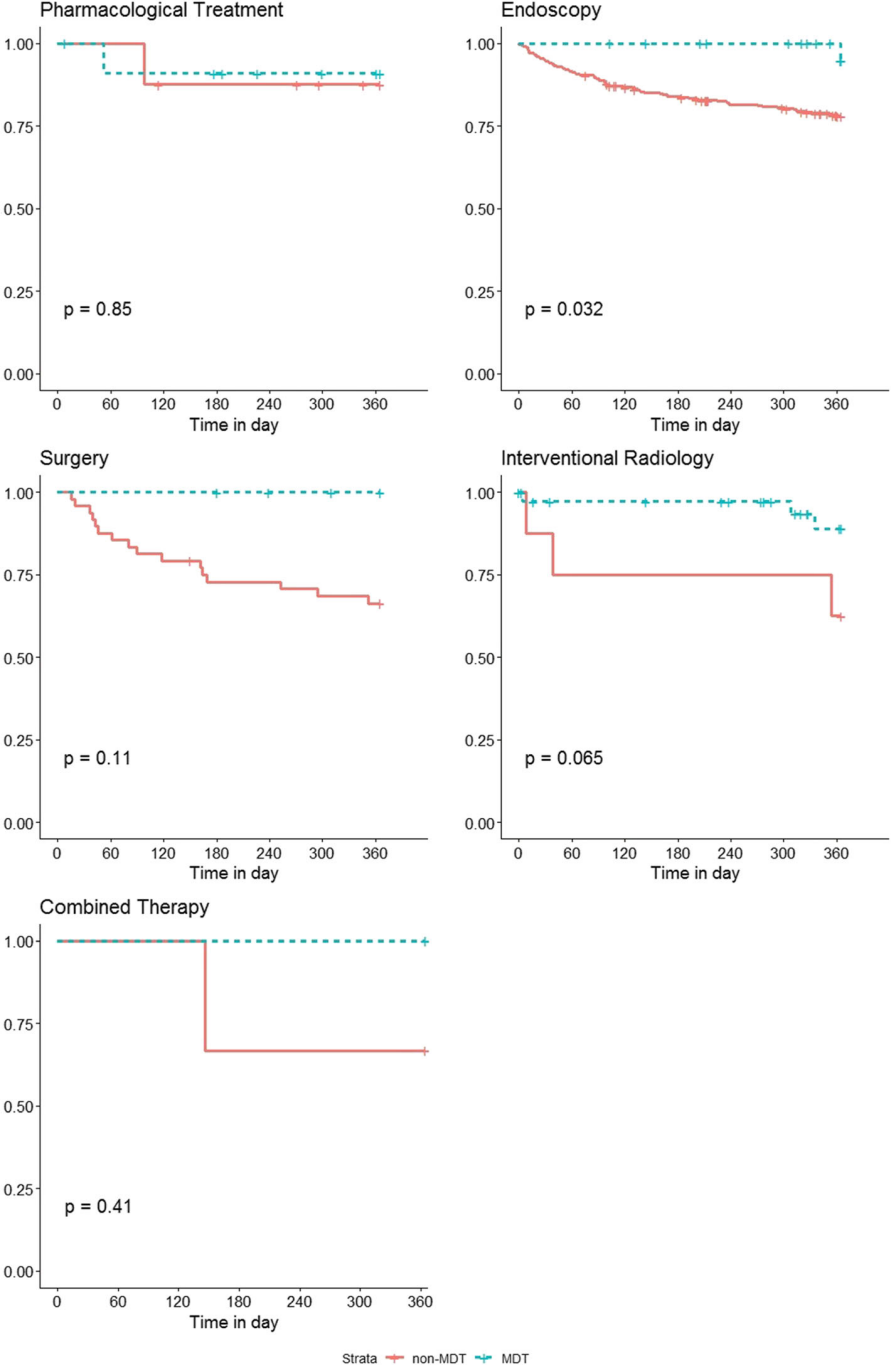
Subgroup analysis of different treatment methods for gastroesophageal varices

A subsequent subgroup analysis with Kaplan-Meir analysis was performed on different treatment modalities after PSM (Fig. 2). The risk of rebleed was statistically different between MDT and non-MDT patient, who underwent endoscopic treatment for gastroesophageal varices (*p* < 0.05). No difference was noted between the two groups in patients who received pharmacological treatment, surgery, interventional radiology or combined therapy. However, a trend is illustrated in the surgery and interventional radiology groups, nearly breaching statistical significance.

## Discussion

According to the Baveno Consensus, primary prophylaxis for gastroesophageal varices include use of non-selective beta-blockers (NSBB) or endoscopic band ligation (EBL), while cyanoacrylate injection should be considered in patients with concurrent or isolated gastric varices. The TIPS procedure may be recommended for patients at high risk of treatment failure [4]. Although the guideline recommendations are based of highly credible clinical studies with promising evidential strength, clinical encounters may not always be straightforward. Patients often have comorbid conditions, which requires well-rounded consideration and prioritization, especially in a complicated disease such a portal hypertension [8, 9].

To our knowledge, the present study is the first to assess the utility of MDT in the management of gastroesophageal varices secondary to portal hypertension. While many other specialties, especially oncology, have successfully adopted MDT as routine practice for disease management [10, 11]. Since the establishment of MDT clinic at our facility, we have included experts from gastroenterology, interventional radiology, general surgery, diagnostic radiology, diagnostic ultrasound, and consulting pathology to form a joint disease management team, with two scheduled discussion panels every month. Prior to the establishment of MDT, patients with gastroesophageal varices are managed with standardized care in different subspecialties.

**Fig. 3:**
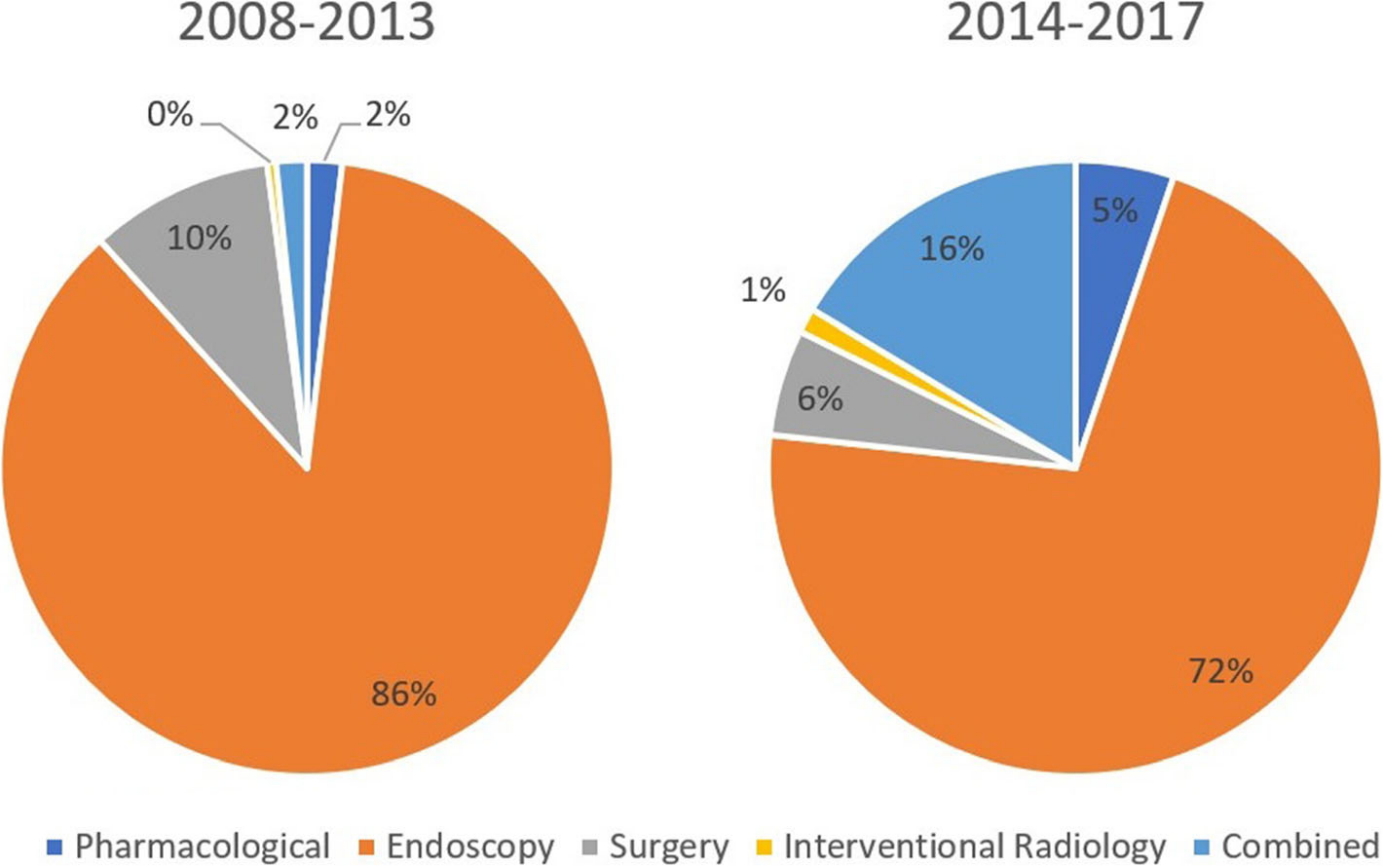
Choice of different treatment modalities for portal hypertension over time

The two study cohorts were compared with propensity score matching. Representative indexes were selected as matching indices. However, treatment modalities were not comparable between the two groups. This can be explained by the development and advancements in interventional radiology in recent years. For instance, the TIPS procedure was not routinely implemented in the past, due to technical challenges and high incidence of hepatic encephalopathy [12]. However, in recent years, availability of self-expandable and drug-eluted stents has remarkably decreased procedure associated complications [13, 14]. Another explanation is that some patients were diverted from endoscopic treatment due to high HVPG measurements [15]. Studies have also shown that splenectomy and devascularization surgery were associated with post-operative portal venous thrombosis, making it a less favorable choice for the treatment of portal hypertension [16]. Therefore, an imbalance in treatment method was noted between non-MDT and MDT patients due to evolving medical techniques and a shift in clinical preference over the past decade (Fig. 3). Comparison between two groups showed no difference in overall survival. However, the rate of variceal bleeding was significantly higher in non-MDT patients before and after PSM. Further subgroup analysis showed the different rebleeding rates amongst patients who received different treatment methods, however study results warrants a larger sample size and longer follow-up.

There are several limitations to our study. First, the sample size for the study is relatively small and the follow-up time is short in the MDT group. A 12 months period may be insufficient to assess the difference in overall mortality between MDT and non-MDT groups. Due to the retrospective nature of the study, not all patients received HVPG measurements, which can accurately reflect disease severity and provide a more reliable matching index for PSM. Lastly, there are many confounding factors that could not be balanced, including different treatment methods for gastroesophageal varices.

## Conclusion

This study is the first to investigate the role of a multidisciplinary team in the management of gastroesophageal varices secondary to portal hypertension. Patients treated based on MDT clinic recommendations had a significantly lower risk for variceal rebleed. MDT-driven disease management is a relatively new concept introduced to clinical practice, especially in the non-oncological arena. Although it is heavily reliant on available resources [17], a multidisciplinary approach allows patient to receive the most well-rounded individualized therapy with consideration beyond medicine. The role of MDT practice has shown promising potential and we look forward to its application to other disease entities.

## Supplementary information

**Additional file 1: Supplementary 1.** MDT Clinic – Case Management. This supplementary file demonstrates an example of case management carried out by a multidisciplinary team specialized in the diagnosis and treatment of portal hypertension.

## Data Availability

Data will be made available upon request to the corresponding author.

## Abbreviations

CTA: Computed tomography angiography
EBL: Endoscopic band ligation
GOV: Gastroesophageal varices
HCC: Hepatocellular carcinoma
IGV: Isolated gastric varices
MDT: Multidisciplinary Team
NSBB: Non-selective beta blockers
PBC: Primary biliary cirrhosis
PSE: Partial splenic embolization
PSM: Propensity matching score
PTVE: Percutaneous transhepatic variceal embolization
PVT: Portal venous pressure
SPSS: Spontaneous portosystemic shunt
TAE: Transarterial embolization
TIPS: TRANSJUGULAR intrahepatic portosystemic shunt
TSE: Total splenic embolization

## References

[1] Seo YS (2018). Prevention and management of gastroesophageal varices. Clin Mol Hepatol.

[2] Bosch J, Garcia-Pagan JC (2003). Prevention of variceal rebleeding. Lancet.

[3] Garcia-Tsao G (2016). Current Management of the Complications of cirrhosis and portal hypertension: Variceal hemorrhage, ascites, and spontaneous bacterial peritonitis. Dig Dis.

[4] de Franchis R (2015). Expanding consensus in portal hypertension: report of the Baveno VI consensus workshop: stratifying risk and individualizing care for portal hypertension. J Hepatol.

[5] Zhang YF, Ji H, Lu HW (2018). Postoperative survival analysis and prognostic nomogram model for patients with portal hypertension. World J Gastroenterol.

[6] Mitchell GK, Tieman JJ, Shelby-James TM (2008). Multidisciplinary care planning and teamwork in primary care. Med J Aust.

[7] Taylor C, Munro AJ, Glynne-Jones R (2010). Multidisciplinary team working in cancer: what is the evidence?. BMJ.

[8] Garcia-Pagan JC, Barrufet M, Cardenas A (2014). Management of gastric varices. Clin Gastroenterol Hepatol.

[9] Ahn SY, Park SY, Tak WY (2015). Prospective validation of Baveno V definitions and criteria for failure to control bleeding in portal hypertension. Hepatology.

[10] Sangiovanni A, Triolo M, Iavarone M (2018). Multimodality treatment of hepatocellular carcinoma: how field practice complies with international recommendations. Liver Int.

[11] Forrest LM, McMillan DC, McArdle CS (2005). An evaluation of the impact of a multidisciplinary team, in a single Centre, on treatment and survival in patients with inoperable non-small-cell lung cancer. Br J Cancer.

[12] Bettinger D, Schultheiss M, Boettler T (2016). Procedural and shunt-related complications and mortality of the transjugular intrahepatic portosystemic shunt (TIPSS). Aliment Pharmacol Ther.

[13] Lo G, Liang H, Chen W (2007). A prospective, randomized controlled trial of transjugular intrahepatic portosystemic shunt versus cyanoacrylate injection in the prevention of gastric variceal rebleeding. Endoscopy.

[14] Lv Yong,Zuo Luo,Zhu Xuan et al. Identifying optimal candidates for early TIPS among patients with cirrhosis and acute variceal bleeding: a multicentre observational study.[J]. Gut. 2019;68:1297–310. 10.1136/gutjnl-2018-317057 30415233

[15] Mura VL (2015). Cirrhosis and portal hypertension: the importance of risk stratification, the role of hepatic venous pressure gradient measurement. World J Hepatol.

[16] Tsamalaidze L, Stauffer JA, Brigham T (2018). Postsplenectomy thrombosis of splenic, mesenteric, and portal vein (PST-SMPv): a single institutional series, comprehensive systematic review of a literature and suggested classification. Am J Surg.

[17] Chinai N, Bintcliffe F, Armstrong EM (2013). Does every patient need to be discussed at a multidisciplinary team meeting?. Clin Radiol.

