# Supplementary material for: The role of a multidisciplinary team in the management of portal hypertension
Source: BMC Gastroenterol. 2020 Apr 3;20:83. doi: 10.1186/s12876-020-01203-4 (PMC7119157; doi:10.1186/s12876-020-01203-4)

**APPENDIX**

**MDT Case Demonstration (Figure A)**

Case Summary: Patient was admitted with a chief complaint of “Hematemesis and melena for 2 days” The patient was diagnosed with chronic hepatitis B (CHB) for 20 years, cirrhosis for 10 years. He initiated antiviral therapy 3 years ago with entecavir. In April 2017, the patient experienced recurrent melena and hematemesis with a progressive decline in hemoglobin. He was treated with conservative therapy at our emergency department and was hemodynamically stable. An endoscopic examination revealed severe gastroesophageal varices with obvious red wales sign (Figure B). HVPG measurement was 13mmHg. A portosystemic CTA revealed portovenous thrombosis and multiple splenic arterial aneurysm (Figure C).

Professor **Pengju Xu** (Diagnostic Radiology): The patient’s radiological studies revealed portal hypertension with formation of collateral circulation. Portal venous thrombosis (PVT) is noted near the bifurcation of main portal vein and the superior mesenteric vein. Multiple splenic arterial aneurysm was also observed, with a largest diameter of 3.3 centimeters.

Professor **Jian Wang** (Gastroenterology): The patient has CHB related portal hypertension, admitted for an initial episode of variceal hemorrhage. EGD revealed a diffused distribution of gastric varices, which is technically difficult for cyanoacrylate injection. Therefore, surgery or interventional radiology should be considered. The patient also has portal venous thrombosis, which requires reasonable anticoagulation therapy with consideration of preventing variceal rebleeding.

Professor **Guohua Hu** (General Surgery): With consideration of the patient’s age and liver function, he is eligible for the Hassab’s procedure (splenectomy and devascularization surgery). However, the patient has multiple splenic arterial aneurysm that are high risk for spontaneous rupture. The aneurysms should be treated with embolization (interventional radiology) prior to surgery.

Professor **Zhiping Yan** (Interventional Radiology): Patient is eligible for the transhepatic portosystemic shunt (TIPS) procedure. Aneurysm embolization can be performed simultaneously with shunt placement (Figure D).

Treatment Options:

1. TIPS procedure with aneurysm embolization (interventional radiology)
2. Aneurysm embolization (interventional radiology) followed by Hassab’s procedure (general surgery)

Patient’s Decision: After consideration, the patient decided opted for treatment option 1.

Figure A. Ongoing MDT Discussion for portal hypertension
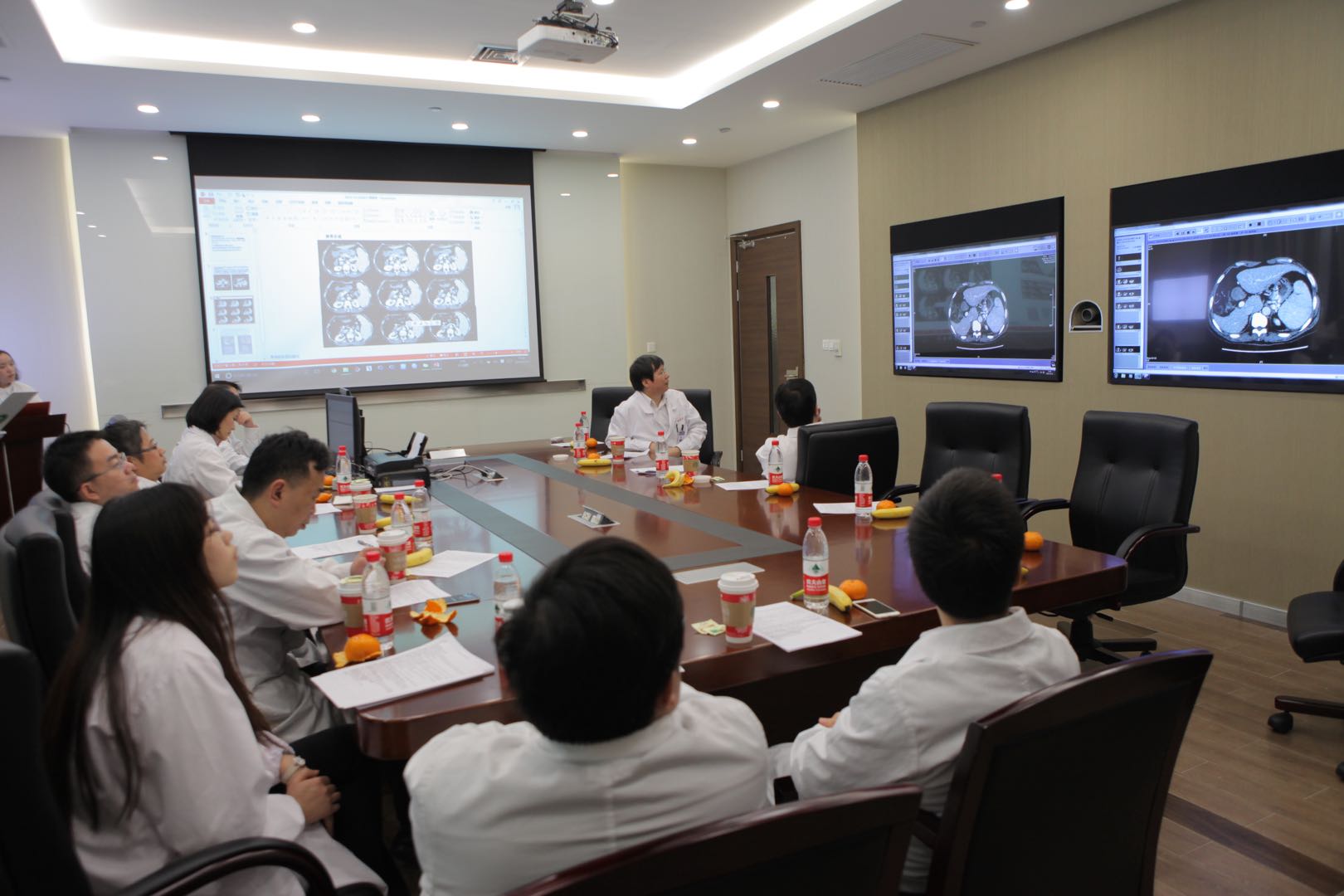


Figure B. Endoscopic examination revealing severe gastroesophageal varices with red wale sign


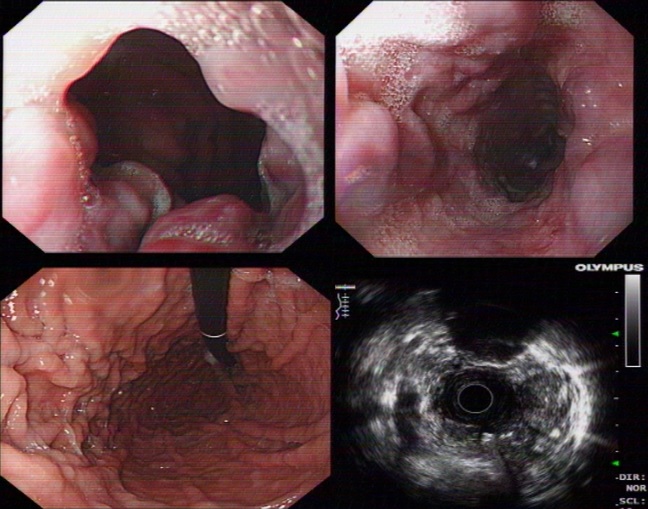


Figure C. Portosystemic CTA with 3D-reconstruction showed portal hypertension with multiple splenic aneurysm


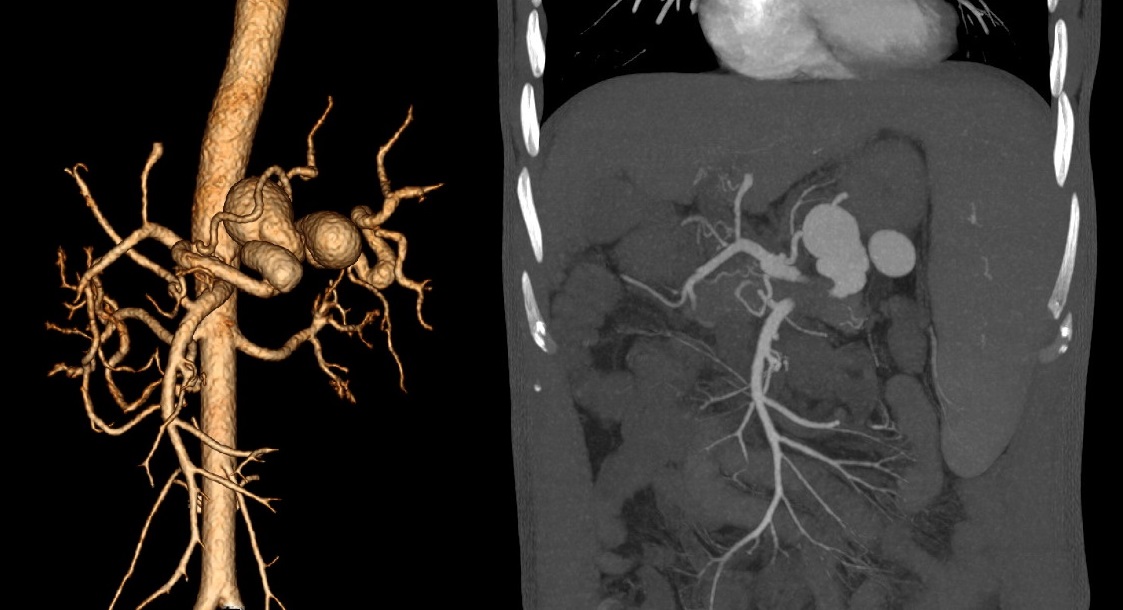


Figure D. Professor Zhiping Yan presenting patient’s radiological studies.
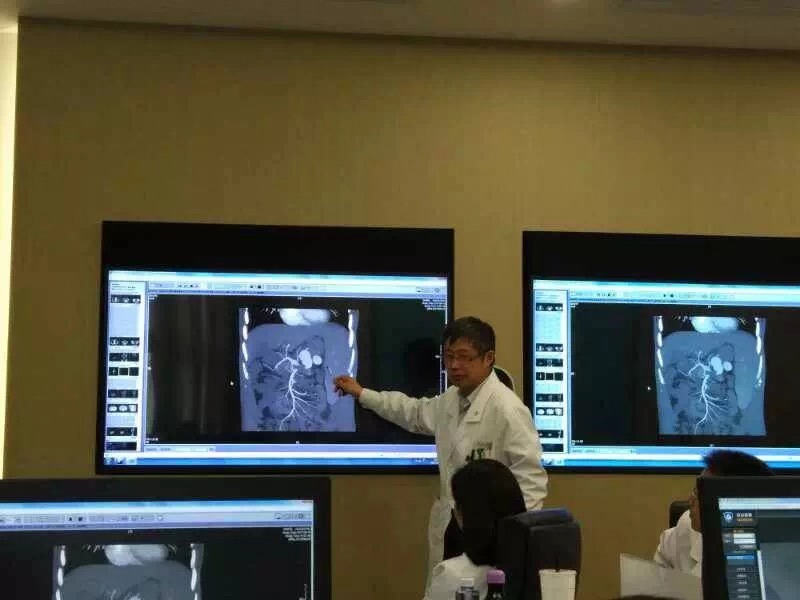

Supplement: Supplementary file 1 — Additional file 1: Supplementary 1. MDT Clinic – Case Management. This supplementary file demonstrates an example of case management carried out by a multidisciplinary team specialized in the diagnosis and treatment of portal hypertension. [file 12876_2020_1203_MOESM1_ESM.docx]
